# Impact of Pretreated Switchgrass and Biomass Carbohydrates on *Clostridium thermocellum* ATCC 27405 Cellulosome Composition: A Quantitative Proteomic Analysis

**DOI:** 10.1371/journal.pone.0005271

**Published:** 2009-04-22

**Authors:** Babu Raman, Chongle Pan, Gregory B. Hurst, Miguel Rodriguez, Catherine K. McKeown, Patricia K. Lankford, Nagiza F. Samatova, Jonathan R. Mielenz

**Affiliations:** 1 Biosciences Division, Oak Ridge National Laboratory, Oak Ridge, Tennessee, United States of America; 2 Computer Science and Mathematics Division, Oak Ridge National Laboratory, Oak Ridge, Tennessee, United States of America; 3 Chemical Sciences Division, Oak Ridge National Laboratory, Oak Ridge, Tennessee, United States of America; 4 BioEnergy Science Center (BESC), Oak Ridge National Laboratory, Oak Ridge, Tennessee, United States of America; 5 Department of Computer Science, North Carolina State University, Raleigh, North Carolina, United States of America; Pacific Northwest National Laboratory, United States of America

## Abstract

**Background:**

Economic feasibility and sustainability of lignocellulosic ethanol production requires the development of robust microorganisms that can efficiently degrade and convert plant biomass to ethanol. The anaerobic thermophilic bacterium *Clostridium thermocellum* is a candidate microorganism as it is capable of hydrolyzing cellulose and fermenting the hydrolysis products to ethanol and other metabolites. *C. thermocellum* achieves efficient cellulose hydrolysis using multiprotein extracellular enzymatic complexes, termed cellulosomes.

**Methodology/Principal Findings:**

In this study, we used quantitative proteomics (multidimensional LC-MS/MS and ^15^N-metabolic labeling) to measure relative changes in levels of cellulosomal subunit proteins (per CipA scaffoldin basis) when *C. thermocellum* ATCC 27405 was grown on a variety of carbon sources [dilute-acid pretreated switchgrass, cellobiose, amorphous cellulose, crystalline cellulose (Avicel) and combinations of crystalline cellulose with pectin or xylan or both]. Cellulosome samples isolated from cultures grown on these carbon sources were compared to ^15^N labeled cellulosome samples isolated from crystalline cellulose-grown cultures. In total from all samples, proteomic analysis identified 59 dockerin- and 8 cohesin-module containing components, including 16 previously undetected cellulosomal subunits. Many cellulosomal components showed differential protein abundance in the presence of non-cellulose substrates in the growth medium. Cellulosome samples from amorphous cellulose, cellobiose and pretreated switchgrass-grown cultures displayed the most distinct differences in composition as compared to cellulosome samples from crystalline cellulose-grown cultures. While Glycoside Hydrolase Family 9 enzymes showed increased levels in the presence of crystalline cellulose, and pretreated switchgrass, in particular, GH5 enzymes showed increased levels in response to the presence of cellulose in general, amorphous or crystalline.

**Conclusions/Significance:**

Overall, the quantitative results suggest a coordinated substrate-specific regulation of cellulosomal subunit composition in *C. thermocellum* to better suit the organism's needs for growth under different conditions. To date, this study provides the most comprehensive comparison of cellulosomal compositional changes in *C. thermocellum* in response to different carbon sources. Such studies are vital to engineering a strain that is best suited to grow on specific substrates of interest and provide the building blocks for constructing designer cellulosomes with tailored enzyme composition for industrial ethanol production.

## Introduction

Plant cell walls consist of several intertwined heterogeneous polymers, primarily composed of cellulose, hemicellulose (substituted xylan), pectin, and lignin. Therefore, the action of several enzymes with diverse catalytic activities is needed in order to efficiently break down and unravel this inherently complex polymer network. The anaerobic, thermophilic, Gram-positive bacterium *Clostridium thermocellum* possesses this diversity in catalytic capability [1], thus making this organism an attractive candidate for lignocellulosic biomass deconstruction and conversion for cellulosic ethanol production [2].

**Figure 1:**
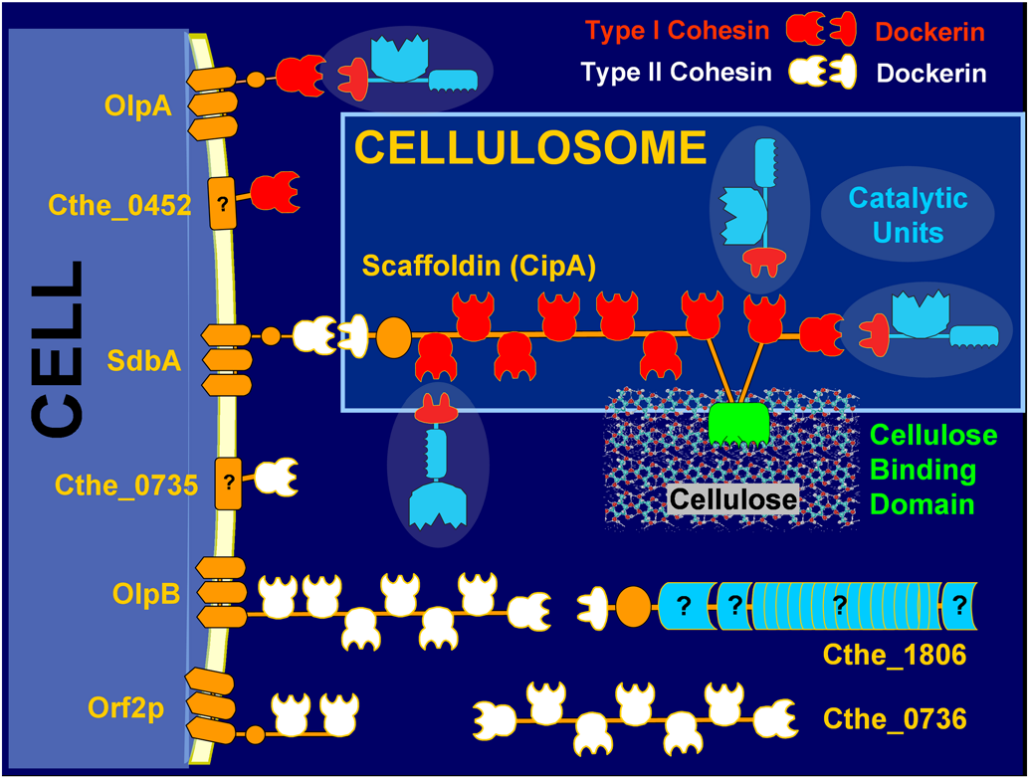
Simplified schematic representation of *Clostridium thermocellum* cellulosomal architecture. CipA is the backbone scaffoldin protein containing 9-Type I cohesins and can accommodate up to 9-Type I dockerin bearing catalytic units. CipA also contains a Type II dockerin for cell-surface attachment *via* anchor proteins and a Cellulose Binding Domain for attachment to the growth substrate. *C. thermocellum* genome encodes five proteins with Type II cohesins, four with S-layer homology domain (SdbA, OlpB, Orf2p and Cthe0735) for cell-surface anchoring of Type II dockerin bearing CipA and one without the SLH domain (Cthe0736). Also shown is the only subunit containing a Type II dockerin (Cthe1806) – (Figure adapted with permission from Carlos Fontes, CIISA, Portugal).

*C. thermocellum* has one of the fastest known growth rates on crystalline cellulose, the major component in plant biomass [3]. High efficiency cellulose hydrolysis is aided by the cell surface attached multienzyme protein complex termed the cellulosome [4], [5], [6]. The cellulosome consists of a primary non-catalytic scaffoldin unit (CipA) that can accommodate as many as nine catalytic units [7]. The catalytic units are non-covalently attached to the scaffoldin *via* the high affinity Type I interaction between dockerin domains borne by the catalytic units with the cohesins on the scaffoldin [8], [9]. In turn, the entire scaffoldin with bound subunits is attached to the cell surface *via* the high affinity Type II interaction between the dockerin domain of CipA and the cohesin(s) borne by the anchoring proteins (OlpB, SdbA, Orf2p) [10]. The scaffoldin and several of the catalytic units also have carbohydrate-binding modules that aid in attachment of the cellulosome directly to the growth substrates to form a cell-cellulosome-substrate tri-complex (see schematic in Figure 1).

Over the past two decades, more than two dozen cellulosome-related genes have been cloned and sequenced. Several cellulosome proteins have been discovered and biochemically characterized based on their substrate-specific activities. Some of the catalytic components possessed diverse hydrolytic activities ranging from exoglucanases (CelS, CbhA, CelK) to endoglucanases (CelA, CelF, CelN, CelB), xylanases (XynA/U, XynY, XynZ), and other hemicellulases (LicB, ChiA), and some proteins contained multiple catalytic modules (CelH, CelJ). [11], [12], [13], [14], [15], [16], [17], [18], [19], [20], [21], [22], [23], [24], [25], [26].

Recent genome sequencing efforts have identified more than 70 dockerin-containing proteins and therefore, potentially cellulosome-related subunits encoded in the genome of *C. thermocellum* 27405 ([27], http://pfam.sanger.ac.uk/ [28]; http://www.cazy.org/ [29]; http://genome.ornl.gov/microbial/cthe/). Genome sequence analysis has revealed the presence of several open reading frames (ORFs) encoding previously unknown proteins in *C. thermocellum* from different families of glycoside hydrolases, carbohydrate esterases, pectin lyases, and two serine protease inhibitors.

With the genome sequence available, three recent studies have utilized mass spectrometry-based methods to identify and experimentally confirm the expression of a number of new cellulosomal proteins. Zverlov et al. [27] used two-dimensional electrophoresis to separate the cellulosomal proteins isolated from cellulose-grown *C. thermocellum* F7 and identified 13 proteins, using Matrix Assisted Laser Desorption and Ionization-Time of Flight (MALDI-TOF) mass spectrometry. More recently, the same team used MALDI-TOF/TOF mass spectrometry to identify 32 components across four different cellulosomal samples isolated from *C. thermocellum* 27405 cultures grown on cellulose, cellobiose, cellulose+xylan, and barley beta-glucan [30].

The most comprehensive proteomic study to date by Gold and Martin [31] employed a metabolic isotope labeling strategy in conjunction with Liquid Chromatography-Tandem Mass Spectrometry (LC-MS/MS) mass spectrometry to estimate quantitative changes in expression patterns of *C. thermocellum* 27405 cellulosomal subunits during growth on cellulose and cellobiose. Qualitatively, 41 cellulosomal components were identified between the two samples, the highest number of experimentally verified subunits thus far. Quantitatively, the authors reported the increased expression of the anchor protein OlpB, exoglucanases CelS and CelK, and the GH9 endoglucanase CelJ, and lowered expression of endoglucanases from glycoside hydrolase families GH8 (CelA) and GH5 (CelB, CelE, CelG) and hemicellulases (XynA, XynC, XynZ and XghA) during growth on cellulose, as compared to cellobiose-grown cellulosomes. Based on these results, the authors suggested a novel distinction in the regulation of GH5 and GH9 endoglucanases.

Other studies have also demonstrated a growth rate and/or carbon source dependent regulation of cellulolytic activity and cellulosomal gene expression in *C. thermocellum* [8], [32], [33]. Specifically, transcript levels of the major exoglucanase, *celS* and endoglucanase genes from GH9 (*celD*) and GH5 (*celB*, *celG*) families have been shown to increase at either low growth rates or in the presence of crystalline cellulose [32], [33], [34], [35], [36], [37]. A similar trend in expression has also been reported for the scaffoldin, CipA and cell-surface anchoring proteins OlpB and Orf2p but not SdbA. However, the underlying regulatory mechanisms for these observations are not well understood. It has been hypothesized that *C. thermocellum* down-regulates the expression of energy-intensive cellulases in the presence of alternate readily metabolizable substrates such as cellobiose *via* catabolite repression [33], [38]. In support of this idea, recently the sugar laminaribiose was identified as an inducer (by inhibiting binding of the negative regulator GlyR3) of the *celC* gene cluster encoding non-cellulosomal enzymes in *C. thermocellum* [39].

While the above studies have provided valuable insights on cellulosomal gene regulation and contributed significantly to identifying the cellulosomal composition, the field would benefit from further detailed investigations. For example, more than 20% of these cellulosomal proteins have domains with no assigned function [2]. Most cellulosomal composition and expression studies have only investigated growth on two model substrates (crystalline cellulose and cellobiose) with the exception of the work by Zverlov and Schwarz [30] which also included barley beta-glucan and xylan in combination with cellulose. While the recent quantitative proteomics study by Gold and Martin has offered a comprehensive look at the cellulosome composition and subunit expression profile, the comparison was only between cellulose- and cellobiose-grown cultures. Additional research is needed for experimental verification of more than 40% of the currently hypothetical cellulosomal proteins as their expression may require the presence of other substrates in the medium for induction.

Therefore, in this study, we investigated qualitative and quantitative changes in cellulosome composition of *C. thermocellum* during growth on a wide variety of growth substrates ranging from crystalline cellulose (Avicel), amorphous cellulose (Z-Trim®), and cellobiose to combinations of cellulose with pectin and xylan. Most importantly, we investigated the cellulosomal expression profile during growth on dilute-acid pretreated switchgrass, a natural biomass substrate for cellulosic ethanol production. Quantitative proteomics (^15^N metabolic labeling coupled with LC-MS/MS) was used to measure substrate-specific changes in cellulosome composition in controlled replicate fermentations. By examining cellulosome expression of *C. thermocellum* during growth on real biomass and multiple combinations of model substrates, we aimed to uncover regulation patterns of cellulosome catalytic subunits by comparing and correlating their expression across these substrates. We hypothesize that the expression and functions of many candidate cellulosomal genes could potentially be ascertained under these complex substrate conditions.

## Materials and Methods

### Fermentation

*C. thermocellum* ATCC 27405 was a gift from Prof. Herb Strobel at the University of Kentucky, Lexington, KY. Fermentations were conducted in 3 L BioStat B jacketed glass fermentors (Sartorius BBI, Inc.) with 2 L working volume of MTC medium at 58°C [33]. Fermentors with media containing only the carbon source were sparged with ultra-high purity nitrogen and vigorously agitated overnight. On the next day, the rest of the media components were added and sparged for an additional 2–3 hrs with nitrogen. A 10% v/v inoculum of cultures pre-adapted on the various substrates in bottles was used to inoculate the fermentors and the gas inlet and exhaust were clamped after inoculation. Samples were taken at regular intervals for protein analysis of pellet and supernatant fractions and HPLC analysis of metabolites. The supernatant protein was estimated using the Bradford assay. Growth was monitored based on increase in the pellet protein concentration. Briefly, cells were lysed in NaOH/SDS solution, cell debris were pelleted and removed, and protein concentration in the supernatant was estimated using the BCA assay. Metabolite analysis was performed using a LaChrom Elite system (Hitachi High Technologies America, Inc.) equipped with a refractive index detector (Model L-2490). Metabolites were separated at a flow rate of 0.5 ml/min in 5 mM H2SO4 using an Aminex HPX-87H column (Bio-Rad Laboratories, Inc.).

### Growth Substrates

Fermentation substrates include cellobiose (Cb, C7252, Sigma Chemical Co., St. Louis, MO), amorphous cellulose (ZT, Z-Trim® dietary fiber containing 60% amorphous cellulose, 16% hemicellulose; ztrim.com), crystalline cellulose (C, Avicel, FMC, PH105, referred to as cellulose hereafter), xylan from oat spelts (X, substituted hemicellulose, X0627, Sigma Chemical Co., St. Louis, MO), pectin from apple (P, P8471, Sigma Chemical Co., St. Louis, MO), and dilute-acid pretreated switchgrass (*Panicum virgatum*; SWG). Switchgrass was pretreated with dilute sulfuric acid using a SUNDS reactor at the National Renewable Energy Laboratory [40]. In mixed substrate fermentations containing cellulose-pectin (C-P), cellulose-xylan (C-X) or cellulose-pectin-xylan (C-P-X), 5 g/L total carbon was added in a weight ratio of 3∶2 or 3∶1∶1, respectively. In the case of switchgrass, 5 g/L dry weight of the pretreated material was used, which had a composition of 50% glucan, 8% xylan and 24% insoluble lignin, based on wet chemistry analysis performed by National Renewable Energy Laboratory as per ASTM E1758-01 method, as part of the BioEnergy Science Center (BESC; http://bioenergycenter.org/) research efforts. In order to obtain ^15^N labeled cellulosomes, *C. thermocellum* was grown on crystalline cellulose in MTC medium containing ^15^N labeled urea and ammonium chloride (Cambridge Isotope Laboratories Inc., Andover, MA) in both the inoculum bottles and fermentors.

### Cellulosome Isolation

Cellulosomes were isolated from cell-free broth from fermentations using the affinity digestion method [41]. Briefly, cultures were spun down and the cell-free broth was incubated with phosphoric-acid-swollen-cellulose (100 mg per liter of cell free broth) overnight at 4°C for cellulase binding to cellulose. On the following day, amorphous cellulose with bound enzymes was spun down and resuspended in 20 mL dialysis buffer (50 mM Tris, 50 mM CaCl_2_, 50 mM DTT, pH 7.0). The amorphous cellulose suspension with bound cellulases was dialyzed in membrane bags (regenerated cellulose, SpectraPor, 6–8 kDa cut-off) at 60°C against 2 L of deionized water to initiate amorphous cellulose degradation by the enzymes. Deionized water was changed every ∼60 mins to avoid inhibition of cellulases by the degradation product, cellobiose. The suspension cleared within 2–4 hrs and a purified cellulase fraction was obtained after further centrifugation of the clarified solution. The total protein concentration of the isolated cellulosome samples was determined with the Lowry assay [42].

### MS/MS Sample Preparation

Every cellulosome sample grown in ^14^N medium was mixed with the reference ^15^N-labeled cellulose cellulosome sample in equal proportions based on the total protein concentration. All mixtures were digested using the following protocol. The proteins were denatured and reduced with 6 M guanidine and 10 mM dithiothreitol (DTT) (D9163, Sigma Chemical Co. St. Louis, MO) at 60°C for 1 h. The samples were then diluted 6-fold with 50 mM Tris, 10 mM CaCl_2_ (pH 7.6), and sequencing grade trypsin (Promega, Madison, WI) was added at 1∶100 (wt∶wt). The first digestion was run for 5 hrs at 37°C and after adding additional trypsin, the second digestion was run overnight at 37°C. Finally, the samples were reduced with 20 mM DTT for 1 h at 60°C and desalted using C18 solid-phase extraction (Sep-Pak Plus, Waters, Milford, MA).

### Quantitative Proteomics Measurement

All samples were examined with liquid chromatography-tandem mass spectrometry (LC-MS/MS) using a five-step, nine-hour, split-phase MudPIT technique [43], [44]. MudPIT measurements were repeated for every sample as technical replication. The samples were loaded via a pressure cell (New Objective, Woburn, MA) onto a 250-µm-I.D. back column packed with 3 cm of C18 reverse-phase resin (Jupiter-3 µm, Phenomenex, Torrance, CA) and 3 cm of strong cation exchange resin (Luna, Phenomenex). The back column was connected to a 15-cm-long 100-*u*m-I.D. C18 reverse-phase PicoFrit column (New Objective) and placed in-line with an Ultimate quaternary HPLC (LC Packings, a division of Dionex, San Francisco, CA). The two-dimensional LC separation was performed with five salt pulses, each of which was followed by a reverse-phase gradient elution. The LC eluent was directly electrosprayed into an LTQ linear ion trap mass spectrometer (ThermoFinnigan, San Jose, CA). Each full scan (400–1700 *m/z*) was followed by three data-dependent MS/MS scans at 35% normalized collision energy with dynamic exclusion enabled. The full scans were averaged from five microscans, and the MS/MS scans were averaged from two microscans.

### Quantitative Proteomics Data Analysis

All MS/MS scans were searched with the SEQUEST program [45] against a *Clostridium thermocellum* ATCC 27405 protein sequence database (http://genome.ornl.gov/microbial/cthe) that contained common contaminants as well as sequence-reversed analogs of each protein for estimation of peptide false discovery rates [46]. The light isotopologs of peptides from the sample proteins were identified using normal amino acid masses in the SEQUEST parameter file, and the heavy isotopologs from the reference proteins were identified using ^15^N-labeled amino acid masses (Enzyme type: trypsin; parent mass tolerance: 3.0; fragment ion tolerance: 0.5; up to four missed cleavages allowed; fully tryptic peptides only). The SEQUEST search results for the two technical replicate measurements of a cellulosome mixture were merged and analyzed by DTASelect 1.9 [47] to yield confident protein identifications. Peptide identifications were filtered by DTASelect based on Xcorr and delCN [Xcorr>1.8 (singly-charged parent ions), >2.5 (doubly-charged parent ions), and >3.5 (triply-charged parent ions); delCN>0.08] and assembled into proteins, retaining duplicate MS/MS scans of a peptide (DTASelect option: −t 0). The abundance ratios for identified proteins in a cellulosome mixture were estimated with the program ProRata 1.1 [48], [49]. Default parameters in ProRata were used, including peptide quantification with a minimum profile signal-to-noise ratio cutoff of 2 for peptide quantification and protein quantification with at least two quantified peptides and a maximum confidence interval width cutoff of 4. Finally, the biological replicate cellulosome mixtures of each comparison were combined with the Combine module of the ProRata program. To filter out proteins with poor quantification reproducibility between the two replicates, only proteins with overlapping confidence intervals in the two biological replicates were retained (Table S1). Proteins with their log_2_ abundance ratios greater than 0.4 or less than −0.4 and confidence intervals excluding 0 were considered as significantly differentially expressed.

### NSAF Calculation

To estimate relative amounts of the various cellulosomal proteins, Normalized Spectral Abundance Factor (NSAF) values [50] were calculated for proteins identified in each LC-MS/MS measurement. For a given protein, the NSAF is the spectrum count for that protein divided by the number of amino acid residues in the protein, divided by this quantity summed over all detected proteins. The spectrum count for a protein is the number of tandem mass spectra assigned to tryptic peptides resulting from digestion of that protein. NSAF calculations included contributions to spectrum count only from unique peptides (those appearing only in a single protein in the predicted *C. thermocellum* proteome). Only peptides identified as ^14^N isotopologs were included in the NSAF calculations. Reported NSAF values are averages from two biological replicates for each sample. Average NSAF values of the components were divided by the average NSAF value for the scaffoldin protein CipA for that sample, and are henceforth referred to as “Weighted NSAF” values.

## Results and Discussion

In order to elucidate substrate-induced changes in cellulosome composition, we grew *C. thermocellum* on pretreated switchgrass and several other model substrates and analyzed the changes in subunit profile of cellulosomes isolated from cell-free broth using quantitative proteomics (^15^N metabolic labeling and LC-MS/MS). Specifically, duplicate *C. thermocellum* fermentations were conducted on dilute-acid pretreated switchgrass (50% glucan, 8% xylan), crystalline cellulose (separate cultures grown in ^14^N- and ^15^N-containing media), cellobiose, Z-Trim® dietary fiber (60% amorphous cellulose, 16% hemicellulose), and combinations of cellulose-pectin (3∶2 wt ratio), cellulose-xylan (3∶2), cellulose-pectin-xylan (3∶1∶1). Cellulosomes were isolated by affinity digestion method from the cell-free broth of late stationary phase cultures during growth on these various substrates. Each cellulosome sample was mixed with an equal proportion of the reference ^15^N-labeled cellulosome sample isolated from cellulose cultures for differential comparison analysis by mass spectrometry.

### Fermentation

**Figure 2:**
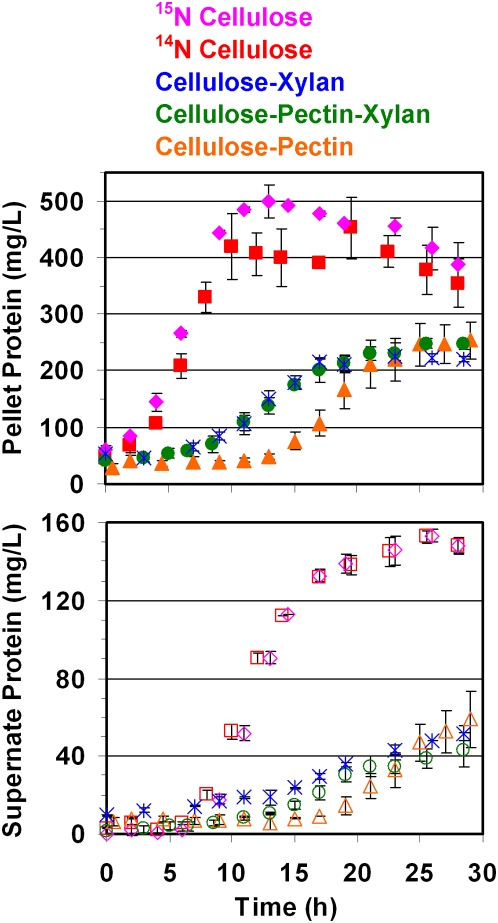
*Clostridium thermocellum* growth profile on various substrates, based upon protein levels. Pellet protein (top panel) and supernatant protein (bottom panel) profiles of *Clostridium thermocellum* during growth on various biomass carbohydrates, as estimated by BCA and Bradford protein assays, respectively. Data represented is the average of two biological replicate fermentations of *C. thermocellum* in minimal medium containing 5 g/L total of the following carbon substrates: Cellulose with ^15^N labeled nitrogen source, Cellulose with ^14^N labeled nitrogen source, Cellulose-Xylan (3∶2 weight ratio), Cellulose-Pectin-Xylan (3∶1∶1), and Cellulose-Pectin (3∶2). Data is not shown for Z-Trim®, pretreated switchgrass and cellobiose fermentations.

In fermentations containing crystalline cellulose, either alone or in combination with pectin, xylan or both, the overall biomass yield, based on total cellular protein levels, was proportional to the amount of cellulose present in the medium (Figure 2). This is not surprising, as *C. thermocellum* cannot grow on xylan or pectin monomers. ^14^N and ^15^N labeled cellulose fermentations yielded similar protein levels, while the mixed substrates supported lower growth rates and total yield with a growth lag. Growth on a blend of 40% pectin 60% cellulose (w/w) showed significantly delayed growth compared to other mixed substrate mixtures (Figure 2).

HPLC analysis of metabolite production (Table 1) revealed an average ethanol+acetate combined yield of 0.37–0.50 g per g of starting glucan during growth on various substrates with the lowest yield on pretreated switchgrass. However, wet chemistry analysis of spent biomass from switchgrass fermentations revealed the presence of unconsumed glucan. Hence, the metabolite yield calculated based on the starting glucan is low due to incomplete conversion of all the glucan in switchgrass fermentations. The acetate to ethanol ratio ranged from 0.9 on crystalline cellulose to 1.88 on pretreated switchgrass (Table 1). Increased acetate concentration in pretreated switchgrass fermentations and Z-Trim® (acetate: ethanol = 1.5; Table 1) may be due to their hemicellulose content (8% and 16% xylan respectively) since acidic deacetylation of hemicellulose leads to increased levels of acetic acid in the medium.

**Table 1:** Acetate and ethanol production during growth on pretreated switchgrass and other biomass carbohydrates.

Substrate	Amount of Substrate added	*Peak Acetate Produced (g/L)	*Peak Ethanol Produced (g/L)	Peak Acetate: Ethanol Ratio	Yield [g acetate+ethanol)/g (glucan)]
^15^N Cellulose	5 g/L	1.17±0.041	1.34±0.045	0.87	0.50
^14^N Cellulose	5 g/L	1.16±0.015	1.27±0.066	0.91	0.49
Cellobiose	5 g/L	1.43	1.02	1.12	0.49
Z-Trim®	5 g/L; ∼60% glucan**	0.81±0.122	0.54±0.047	1.50	0.45
Cellulose-Xylan	5 g/L total; 3∶2 wt ratio	0.71±0.106	0.62±0.101	1.15	0.44
Cellulose-Pectin	5 g/L total; 3∶2 wt ratio	0.69±0.033	0.49±0.052	1.41	0.39
Cellulose-Pectin-Xylan	5 g/L total; 3∶1∶1 wt ratio	0.60±0.113	0.54±0.106	1.11	0.38
Pretreated Switchgrass	5 g/L dry wt; ∼50% glucan**	0.60±0.163	0.32±0.062	1.88	0.37

* Data reported is average from duplicate fermentations on each substrate except cellobiose.

** Glucan content estimated by wet chemistry analysis.

**Figure 3:**
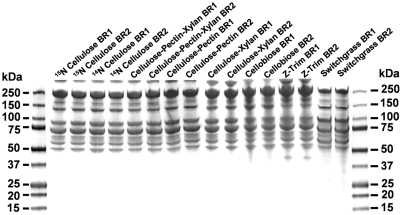
*Clostridium thermocellum* cellulosomal protein profile during growth on various substrates, analyzed by protein gel electrophoresis. SDS-PAGE (4–20% Tris-HEPES-Glycine gel, coommassie stain) separation of *Clostridium thermocellum* cellulosomal fractions isolated using the affinity digestion method from cell-free broth of duplicate fermentations during growth on pretreated switchgrass and other biomass carbohydrates. BR1, 2 = Biological Replicate 1, 2, respectively.

Under each growth condition, there was a significant increase over time in the protein amount present in the cell-free growth medium (Figure 2), beginning at approximately 7.5 hours when grown on cellulose and later for the slower growing cultures. To demonstrate that this increase was due to cellulosomes being released into the medium, as reported earlier [51], cellulases present in the supernatant of late stationary phase cultures for each substrate were captured and isolated using the affinity digestion method. The isolated proteins were examined by SDS PAGE separation which yielded a very consistent profile representative of cellulosome components [32] between the biological replicates for each of the different substrate growth conditions. Substrate-related changes in cellulosomal subunit profiles were not readily apparent based on the SDS-PAGE gel pattern (Figure 3).

### Proteomics Analysis

**Figure 4:**
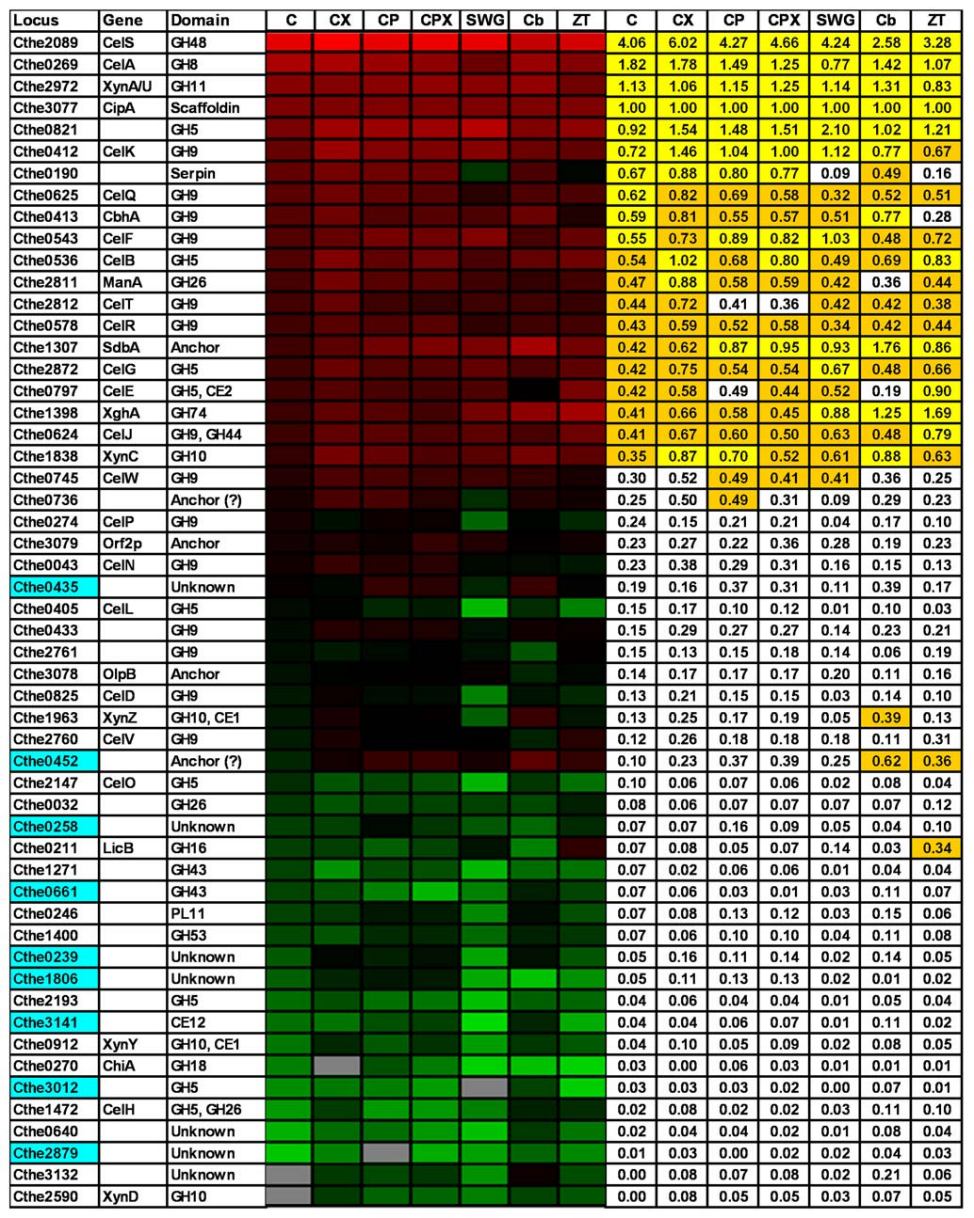
Weighted-Normalized Spectral Abundance Factor (Weighted-NSAF) of cellulosomal components in mass spectrometry analysis. Weighted-NSAF data for ^14^N isotopologs across seven different samples is arranged in descending order of values for cellulose sample. For each cellulosomal sample, the average NSAF value of the various components was divided by the value for the scaffoldin protein CipA to obtain weighted-NSAF values. Heat plot representation shows weighted-NSAF distribution (Red, highest; Green, lowest) of 54 cellulosomal subunits. The proteins with top 10, and the next 10, highest weighted-NSAF values in each sample are highlighted in Yellow and Orange, respectively. Locus entries of newly detected cellulosome components are highlighted in Blue. Substrate legend: C = Cellulose, CX = Cellulose-Xylan, CP = Cellulose-Pectin, CPX = Cellulose-Pectin-Xylan, SWG = Pretreated Switchgrass, Cb = Cellobiose, ZT = Z-Trim®.

**Figure 5:**
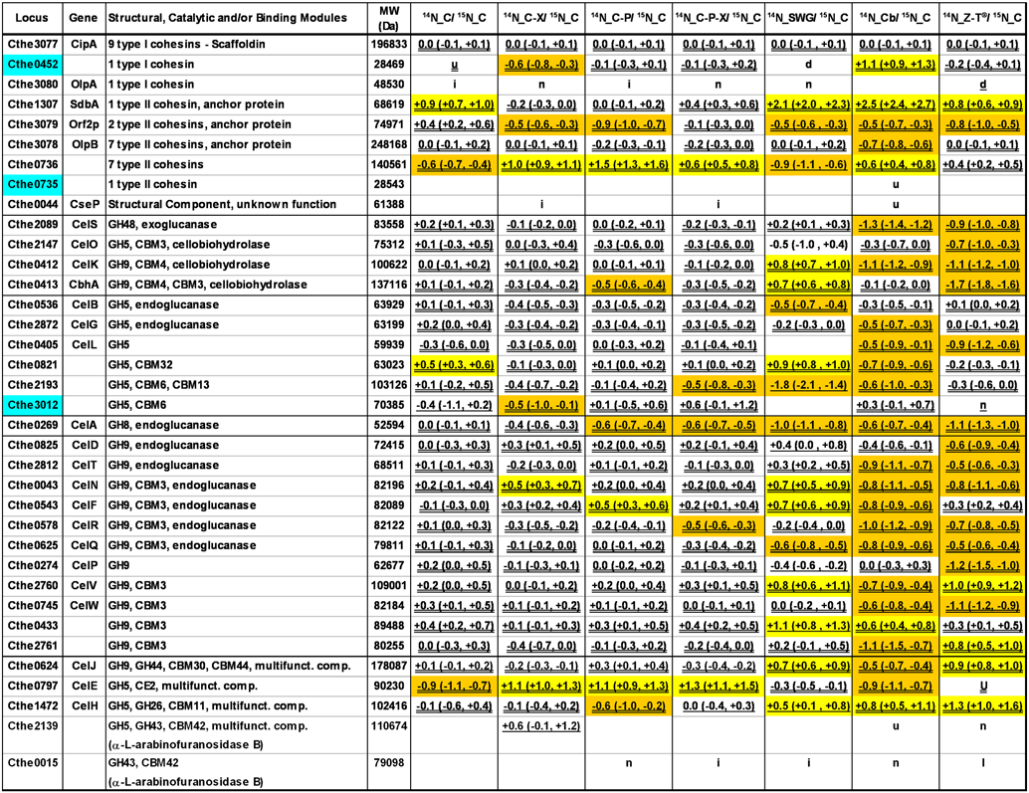
Quantitative changes in *Clostridium thermocellum* cellulosome composition in response to carbon substrate – Part I *(see* *Figure 6* *caption).*

**Figure 6:**
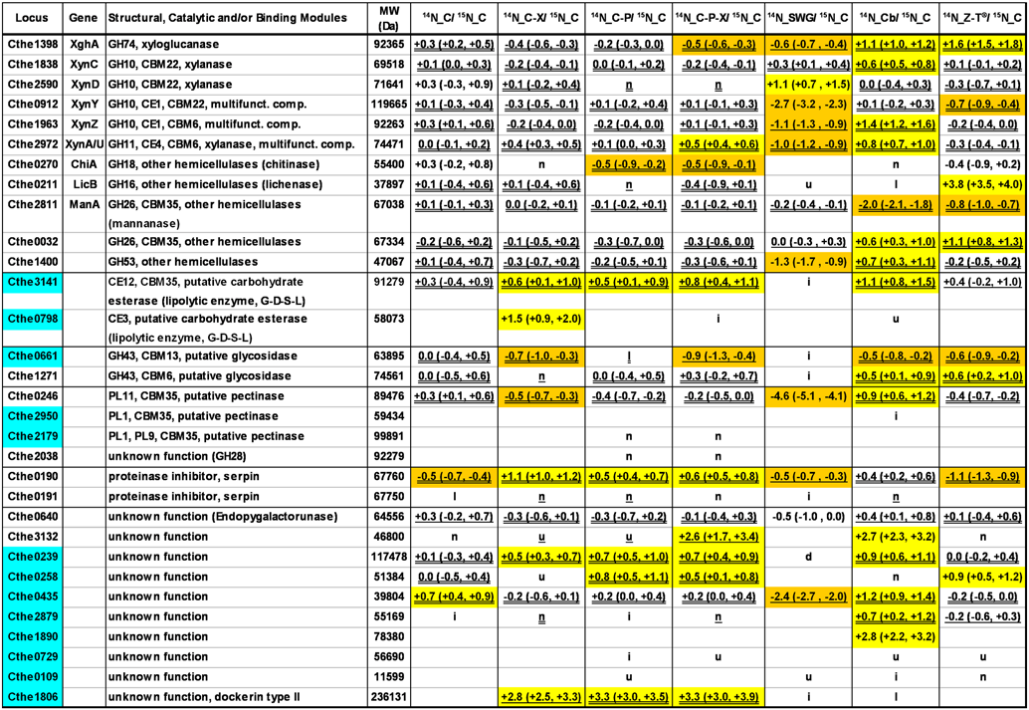
Quantitative changes in *Clostridium thermocellum* cellulosome composition in response to carbon substrate – Part II. Quantitative data shown is combined from two biological and two technical replicates and expressed as Log_2_Ratio (LowerCI, UpperCI) of cellulosomal component X during growth on substrate Y over that in ^15^N-labeled Cellulose cellulosome sample. Substrate key: C = Crystalline Cellulose (with ^14^N or ^15^N labeled nitrogen source), C-X = Cellulose-Xylan (5 g/L total in 3∶2 weight ratio), C-P = Cellulose-Pectin (3∶2), C-P-X = Cellulose-Pectin-Xylan (3∶1∶1), SWG = Pretreated Switchgrass (50% glucan, 8% xylan, 24% lignin), Cb = Cellobiose, Z-T = Z-Trim® (60% amorphous cellulose, 16% hemicellulose). Data was normalized to the scaffoldin CipA protein. Locus entries highlighted in Blue have not been observed experimentally prior to this study. Quantitation data highlighted in Yellow (increased expression in Substrate Y) and Orange (decreased expression in Substrate Y) satisfy the cut-off criteria. Criteria for differential expression was based on control comparison of ^14^N- and ^15^N-labeled cellulose cellulosome samples and were, (1) Log_2_Ratio should be >+0.4 or <−0.4 and (2) Upper/Lower Confidence Intervals should exclude Log_2_Ratio = 0. Structural, catalytic and/or binding module information was obtained from the following sources: http://pfam.sanger.ac.uk/; http://www.cazy.org/; http://genome.ornl.gov/microbial/cthe/. Domain key: GH = Glycoside Hydrolase, CE = Carbohydrate Esterase, PL = Polysaccharide Lyase, CBM = Carbohydrate Binding Module. Legend key: MW = Molecular Weight, Log_2_Ratio (LowerCI, UpperCI) - protein was quantified in both biological replicates (BR), with overlapping confidence intervals; Blank - protein not identified or quantified; d - protein quantified in only one BR, but result indicates down-regulation (UpperCI<0, Log_2_ratio<−0.4); D - protein quantified in both BR, but confidence intervals don't overlap. Both confidence intervals indicate down-regulation (UpperCI<0, Log_2_ratio<−0.4); i - identified in one BR, but no quantification result; I - identified in both BR, but no quantification result; n - protein quantified in only one BR, with result indicating no change in expression (−0.4<Log_2_ratio<+0.4); N - protein quantified in both BR, but confidence intervals don't overlap. Both confidence intervals indicate no change in expression (−0.4<Log_2_ratio<+0.4); u - protein quantified in only one BR, but result indicates up-regulation (LowerCI>0, Log_2_ratio>+0.4); U - protein quantified in both BR, but confidence intervals don't overlap. Both confidence intervals indicate up-regulation (LowerCI>0, Log_2_ratio>+0.4); A 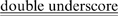 indicates that the protein was identified in both ^14^N and ^15^N forms in both BR; A single underscore indicates that the protein was identified in both ^14^N and ^15^N forms in only one BR; No underscore indicates that we did not identify both ^14^N and ^15^N forms in either BR.

The metabolic labeling strategy coupled with LC-MS/MS technology was used to identify and quantify the cellulosomal proteins using ^15^N-labelled cellulosomes isolated from cellulose cultures as the reference. ^14^N labeled cellulosomes isolated from cell-free broth of duplicate fermentations on seven different substrates were mixed in 1∶1 ratio with ^15^N labeled cellulosomes isolated from cellulose cultures. In total, 14 cellulosome sample mixtures were prepared for analysis by mass spectrometry in technical replicate runs. Protein data from biological and technical replicate LC-MS/MS MudPIT runs were combined and expression differences were normalized based on the CipA scaffoldin protein across different comparisons (Figures 4, 5, 6). The affinity digestion-based cellulosome isolation procedure in effect captures the whole complex, which is built on the scaffoldin protein, thus justifying this type of normalization of data based upon the distribution of cellulosomal proteins on a per scaffoldin basis [27], [31].

### Subunits Identification

In total, mass spectrometry analysis detected 67 cellulosomal proteins between the seven samples (Figures 4, 5, 6), which includes 80% (59/73) of the dockerin module containing proteins and 100% (8/8) of the cohesin containing proteins in *C. thermocellum*, based on genome analysis [27]. Among the different cellulosomes analyzed, cellobiose samples yielded the highest (64) and switchgrass the lowest (53) number of protein identifications; the latter is likely due to the complexity of the growth substrate affecting the quality of the cellulosomal preparation.

We identified 16 new cellulosomal components in this study (Figures 5, 6, highlighted in blue). This represents a 30% (16/53) increase in the total number of subunits identified and biochemically verified to date, including the two recent comprehensive studies by Gold/Martin and Zverlov/Schwarz [30], [31]. Out of the 16 newly detected cellulosomal subunits, 7 proteins were detected under all conditions tested, while others were observed only in a subset of the samples. While many of the newly detected subunits were low abundant proteins, two proteins (Cthe0435 and Cthe0452) appeared to be fairly abundant, based on NSAF (Figure 4). In fact, Cthe0452, a potential anchor protein containing one type I cohesin, was among the 20 proteins with the highest spectral abundance (weighted NSAF) during growth on cellobiose and Z-Trim® (Figure 4).

We did not detect 14 out of 81 predicted cellulosome-related structural and catalytic proteins in *C. thermocellum* under any of the conditions, including the only representative proteins from glycoside hydrolase families GH81, GH2 and GH39 encoded in *C. thermocellum* genome. Among the undetected proteins, Cthe2360 (CelU, GH9) and Cthe3136 (S8, S53 peptidase) have been observed earlier by Zverlov et al [30]. Many of the undetected proteins are encoded by contiguous genes (e.g. *Cthe2137-2138*, *Cthe2194-2195-2196-2197*, and *Cthe2949-2950*) suggesting that these are likely inducible ‘operons’ and, hence, were not expressed due to the lack of their potential ‘inducers’ under the growth conditions tested.

### Subunit Abundance Distribution

Weighted NSAF values were used to determine a rough estimate of the relative abundance of cellulosomal components within the different samples (Figure 4). NSAF values [50] for each protein within a sample were divided by the NSAF for the scaffoldin CipA protein in that sample to yield weighted NSAF values.

The majority (49/67) of the identified proteins were detected under all growth conditions tested although their relative amounts within each sample were different depending on the growth substrate. Based on weighted NSAF data, the 20 most abundant proteins were similar across all the substrates containing crystalline cellulose, but not during growth on Z-Trim® or cellobiose (Figure 4). It appears that the cellulosomal proteins follow the 10–60 or 20–80 law, i.e., the top 10 or 20 most abundant proteins (based on weighted NSAF) account for 60% or 80% of the total cellulosomal protein fraction, respectively. The exoglucanase CelS had the highest spectral abundance under all growth conditions including cellobiose. However, this is in contrast to a previous study which reported xylanases as the most abundant components in cellobiose-grown cellulosomes [31]. This may be due to differences between the NSAF approach that we used and the Protein Abundance Index method used by Gold and Martin.

### Quantitative Proteomics

Relative quantitative expression data for the cellulosomal proteins during growth on pretreated switchgrass and other biomass carbohydrates, as compared to growth on cellulose, are reported in Figures 5, 6. The abundance ratios in quantitative expression data were normalized based on the CipA protein to look at changes in expression of the cellulosomal proteins on per scaffoldin basis across the different growth conditions. The comparison between ^14^N and ^15^N labeled cellulose-grown cellulosome samples served as a control to evaluate biological and technical reproducibility of cellulosomal protein expression analysis and to determine criteria for differential expression. Based on this comparison, proteins with their relative log_2_ abundance ratios greater than 0.4 or less than −0.4 and confidence intervals excluding 0 were considered as significantly differentially expressed.

On average, 49 proteins were quantified in each of the seven comparisons for the different growth substrates (Figures 5, 6). Cellulosome samples isolated from cultures grown on cellobiose, amorphous cellulose (Z-Trim®) and pretreated switchgrass showed the most distinct differences in cellulosomal protein levels (as compared to crystalline cellulose) with 40 (78%), 28 (56%) and 25 (61%) of the quantitated proteins, respectively, showing significant differential expression (Figures 5, 6). Relatively fewer proteins (13–15) were differentially expressed in the case of cellulosomes isolated from cultures grown on cellulose in combination with xylan or pectin, or both. The control comparison between ^14^N and ^15^N cellulose-grown cellulosome samples showed minimal technical and biological variability in cellulosomal protein expression with only 6 of the quantified proteins with significant differential expression based on the criteria described above.

Grouping the proteins based on their structural function or catalytic activity identified growth substrate-related trends in cellulosomal protein expression for structural proteins, exoglucanases, endoglucanases belonging to GH5 and GH9 families, xylanases, and other hemicellulases, as outlined below.

### Structural Proteins

All known proteins containing type I and type II cohesin (Coh I, Coh II) modules in *C. thermocellum* (see Figure 1) were detected in this study, including two proteins, Cthe0452 and Cthe0735, that have not been observed experimentally prior to this study.

Among the Coh I containing proteins, OlpA (with 1 Coh I domain) has been suggested to play an intermediary role in the assembly of the cellulosome complex by binding the catalytic units prior to their transfer and assembly on the scaffoldin CipA (Figure 1) [52], 53. Another protein with a single Coh I, Cthe0452, was detected with significantly higher weighted-NSAF than OlpA (Figure 4) under all growth conditions. This observation may suggest a yet unknown but potentially important role for this protein in the cellulosome assembly process, as has been hypothesized for OlpA. We also observed increased expression of the Cthe0452 protein during growth on cellobiose (Figures 5, 6), as compared to cellulose-grown cellulosomes, in our quantitative proteomics analysis. This may be related to a similar pattern in expression observed for Cthe435, a subunit of unknown function, as the dockerin module on the latter is known to interact specifically with the cohesin on Cthe0452 (Carlos Fontes, personal communication).

The affinity digestion method of cellulosome isolation used in this study is targeted towards the capture of the subunit-laden CipA scaffoldin *via* its cellulose-binding module. Therefore, the detection of type II cohesin containing proteins that anchor the scaffoldin CipA to the cell surface (Figure 1) supports the hypothesis that the detachment of intact cellulosomes from the cell surface in mature cultures of *C. thermocellum* is possibly achieved by proteolytic cleavage of the anchor proteins [10], [51].

Among the five proteins with type II cohesin domains in *C. thermocellum* (Figure 1), except Cthe0735 (with 1 Coh II domain) which was detected only during growth on cellobiose, all other proteins were detected under all growth conditions (Figures 4, 5, 6). In general, the relative spectral abundance of Coh II containing anchor proteins within each sample was inversely proportional to the number of cohesin modules borne by them, with SdbA (with 1 Coh II domain) being the most spectrally abundant and OlpB (with 7 Coh II domains) or Cthe0736 (with 7 Coh II domains), the least abundant across the different cellulosomal preparations (based on weighted-NSAF data, Figure 4). These results are in contrast to earlier reports of OlpB or Orf2p being the most prominent anchor protein during growth on cellulose or cellobiose, respectively [31], [35]. Dror et al., based on transcript levels, reported a 10-fold excess in the number of cohesins available on the anchor proteins for attaching the scaffoldin CipA to the cell surface under conditions of low growth rate [35]. In this study, we estimated a 3.5–6 fold excess of cohesins over CipA at the protein level, based on the spectral abundance, during growth on different substrates (Figure 4).

However, it should be noted that the spectral abundance of a protein is influenced by several factors including the detectability of its peptides. It is known that the structural proteins of the cellulosome, namely the anchors and scaffoldin, are often glycosylated to protect the complex against proteolysis [54]. This leads to differences in their cleavability by trypsin and also the detectability of the resulting peptides due to mass-shifts resulting from glycosylation, thus influencing the spectral abundance. Therefore, studies involving absolute quantitation of these proteins are needed to investigate further the observed trends in type II cohesion-dockerin ratios, which would also provide insight into the plasticity or elasticity of the cellulosome complex.

SdbA exhibited the second highest weighted-NSAF in mass spectrometry analysis of cellobiose-grown cellulosomes (Figure 4). Correspondingly, quantitative proteomics analysis also showed a >5-fold increase in expression of SdbA during cellobiose growth as compared to cellulose-grown cellulosomes (Figures 5, 6), consistent with a previous study [31]. Higher levels of SdbA were also observed under conditions of relatively slow growth on pretreated switchgrass and fast growth on Z-Trim (Figures 5, 6) and thus presumably regulated in a growth-rate independent manner as reported earlier [35]. On the other hand, a growth rate and/or carbon source dependent regulation has been reported for the genes encoding the anchor proteins, OlpB and Orf2p, at the transcript level [35]. Consistent with these results, we observed lower levels of the OlpB protein under fast growing conditions on cellobiose (Figures 5, 6), which has also been reported by Gold and Martin [31]. However, no such correlation was observed for Orf2p (Figures 2, 5, 6).

Interestingly, recent genome sequencing revealed the presence of another type II cohesin containing protein, Cthe0736 (with 7 Coh II domains), but the protein lacks the surface layer homology (SLH) domain needed for cell surface anchoring. This suggests the potential presence of “free” non-cell attached cellulosomes, formed *via* the type II interaction between the scaffoldin CipA and the Cthe0736 protein, in *C. thermocellum*. The “free” cellulosomes could aid in targeting catalytic subunits, involved in hydrolysis of non-cellulosic material, to surfaces of complex substrates for exposing the preferred substrate of cellulose for hydrolysis and consumption. Alternatively, these “free” cellulosomes may yet remain cell-associated (if not cell-attached) through their integration in the glycocalyx matrix of the polycellulosomal complexes. *C. thermocellum* is known to form protuberant structures of polycellulosomes on the cell surface consisting of several hundred cellulosomes with masses up to 100 MDa [51]. In this study, Cthe0736 showed increased expression during growth on all substrates, except pretreated biomass, as compared to cellulose (Figures 5, 6). Further research is needed to understand the observed trends in expression and to unravel the function and regulation patterns of this novel protein.

### Exoglucanases

All four known cellulosomal exoglucanases in *C. thermocellum* belonging to families GH48 (CelS), GH9 (CelK, CbhA) and GH5 (CelO) were detected in this study. CelS was the most spectrally abundant protein, while CelO was a relatively minor component, in the cellulosomal preparations irrespective of the growth substrate (based on weighted-NSAF data; Figure 4). Our results confirm earlier reports that CelS is the major component in *C. thermocellum* cellulosomes [27], [55], [56], [57]. The four exoglucanases accounted for 18–30% of the total spectral abundance in the cellulosomal fractions under the different growth conditions, with the least proportion during growth on cellobiose and Z-Trim (Figure 4).

Correspondingly, quantitative proteomics showed lower expression of all four exoglucanases during growth on Z-Trim® (60% amorphous cellulose) than on cellulose. CelS and CelK also showed decreased expression during growth on cellobiose, as compared to growth on cellulose, with no significant difference in the expression of CbhA or CelO (Figures 5, 6). Previous studies have reported a growth rate dependent regulation of *celS* gene with reduced levels of gene expression at both transcript [34], [37] and protein level [34] in cellobiose-grown cells, as compared to crystalline cellulose-grown cells. In this study, the observed trend of lower CelS protein expression under fast growing conditions on Z-Trim and cellobiose (data not shown) is consistent with this type of regulation. Moreover, CelS has higher activity on amorphous cellulose than crystalline cellulose [57], which might explain the need for lower levels of CelS during growth on Z-Trim. Recently, Gold and Martin also reported a similar trend in expression of CelS and CelK proteins with decreased levels during growth on cellobiose, as compared to cellulose [31].

On the other hand, the duplicated family 9 cellobiohydrolases, CelK and CbhA, both were expressed at higher levels during growth on pretreated switchgrass, as compared to cellulose (Figures 5, 6). Among the four cellulosomal exoglucanases in *C. thermocellum*, the GH9 exocellulases attack the cellulose chain from the non-reducing end whereas CelS and CelO attack from the reducing end of the chain [19], [26]. Therefore, the increased expression of CelK and CbhA suggests an enhanced need for exo-exo synergy between these two classes of exocellulases, with different specificities, in cells grown on natural plant biomass to attack the cellulose in this complex substrate from both reducing and non-reducing ends [58].

### Endoglucanases

All known cellulosomal endoglucanases in *C. thermocellum* belonging to glycoside hydrolase families GH5 (9 proteins in total), GH8 (1) and GH9 (12 in total) were detected in this study, with the exception of the GH9 subunit CelU (Cthe2360) (Figures 4, 5, 6). We also experimentally confirmed a new GH5 subunit (Cthe3012) as a cellulosomal component. Multimodular cellulosomal proteins containing other catalytic modules, in addition to GH5 and GH9 domains, are grouped separately in Figures 5, 6. The following discussion assumes endoglucanase activity for all previously uncharacterized GH5 and GH9 cellulosome components.

In general, CelA (GH8) and the recently discovered GH5 subunit (Cthe0821) were the two most spectrally abundant endoglucanases (based on weighted-NSAF data, Figure 4) in cellulosomal preparations, during growth on various substrates. This is consistent with previous reports of CelA being the major endoglucanase in *C. thermocellum* cellulosomes [3]. However, cellulosomes isolated from cultures grown on pretreated switchgrass were an exception and contained Cthe0821 and CelF (Cthe0543, GH9) as the most spectrally abundant endoglucanase components (Figure 4). In general, GH5 endoglucanases, Cthe0821, CelB, CelG, and CelE and GH9 endoglucanases, CelQ, CelF, CelT, CelR, CelW and CelJ were among the top 20 catalytic components with the highest weighted-NSAF in all cellulosomal preparations, irrespective of the growth substrate.

Quantitative proteomics analysis revealed a general trend toward decreased expression of GH5 endoglucanases during growth on cellobiose, as compared to cellulose (Figures 5, 6). This is in contrast to a recent study which reported increased expression of GH5 proteins during cellobiose growth [31]. However, our results are consistent with a transcript-level study by Dror et al. which showed a growth-rate dependent regulation of CelB and CelD genes with decreased transcript levels in cellobiose cultures, as compared to cellulose [36]. On the other hand, no significant changes in expression of GH5 endoglucanases were observed, with the exception of decreased CelL expression, during growth on Z-Trim® (Figures 5, 6). Quantitative proteomics also showed increased expression of the GH5 endoglucanase, Cthe0821, in switchgrass-grown cellulosomes, but lower levels of CelB and Cthe2193 as compared to cellulose (Figures 5, 6).

Endoglucanases belonging to the GH9 family also showed a trend toward decreased expression during growth on cellobiose, as compared to growth on cellulose (Figures 5, 6). These results are broadly in agreement with previous studies which also reported decreased expression of GH9 endoglucanases in cellobiose cultures, both at the transcript [36] and the protein level [31]. In general, GH9 endoglucanases were also expressed at lower levels during growth on Z-Trim®, with the exception of two contiguous genes, Cthe2760 (CelV)-2761, which showed higher expression, as compared to cellulose (Figures 5, 6). These results show that GH9 endoglucanases are specifically down-regulated in the absence of crystalline cellulose in the growth medium. On the other hand, the increased expression of several GH9 endoglucanases, CelN, CelF, CelV and Cthe0433, during growth on pretreated switchgrass (Figures 5, 6) highlights the important role of this family of endoglucanases in the degradation of natural plant biomass.

As discussed above, we observed a differentiation with respect to the expression of GH5, but not GH9, endoglucanases between cellobiose and Z-Trim-grown cultures. While GH9 endoglucanases showed decreased expression during growth on both cellobiose and Z-Trim, GH9 endoglucanases showed decreased expression only during growth on cellobiose (Figures 5, 6). Put another way, while GH9 endoglucanases showed decreased expression in the absence of crystalline cellulose, GH5 endoglucanases showed decreased expression in the absence of cellulose in general, amorphous or crystalline, in the growth medium. Taken together, these results suggest an important role for the GH9 endoglucanases in the decrystallization of crystalline cellulose. We propose that GH9 endoglucanases attack the crystalline surface of cellulose fibrils aiding in the creation of amorphous cellulose regions which become targets for hydrolysis by GH5 endoglucanases.

Among the multimodular proteins, the increased expression of the CelH (Figures 5, 6) during growth on cellobiose, Z-Trim® and pretreated switchgrass may be attributable to the presence of a second GH26 catalytic unit in the protein. During growth on combinations of cellulose, xylan, and pectin, CelE (GH5, CE2 domains) showed increased expression in all three conditions but not during growth on switchgrass, possibly due to the presence of the carbohydrate esterase catalytic unit.

### Xylanases

Among the known cellulosomal xylanases, XynA, XynC, XynZ, and XghA showed higher expression during growth on cellobiose, as compared to cellulose-grown cellulosomes (Figures 5, 6). In fact, xylanases accounted for 22% of the total spectral abundance in the cellulosomal fraction during growth on cellobiose, as compared to 12% during growth on cellulose (based on weighted-NSAF data, Figure 4). These results are consistent with the growth independent regulation of xylanases reported in earlier studies [8], [36] with higher expression of xylanases during growth on cellobiose, as compared to cellulose [31].

*C. thermocellum* cannot grow on pentose sugars [3] derived from catalytic activities of xylanases and other hemicellulases. Therefore, these enzymes are suggested to play a vital role in exposing the preferred substrate, cellulose, in plant cell walls through the degradation of hemicellulose and other polymeric substrates [1]. Interestingly, xylanases showed decreased expression during growth on pretreated switchgrass relative to growth on cellulose (Figures 5, 6), which could be detrimental in unmasking the preferred substrate of cellulose during growth on natural plant biomass. However, it is possible that the residual hemicellulose (8% xylan) in pretreated switchgrass is buried under lignin-cellulose complexes, thus minimizing potential xylanase inductive effect. The lack of pectin in pretreated switchgrass may also explain the significant downregulation (>16-fold) of one of the few pectate-active enzymes (Cthe0246) in *C. thermocellum* during growth on plant biomass versus growth on cellulose.

To date, this study provides the most comprehensive comparison of cellulosomal compositional changes in *C. thermocellum* in response to different carbon sources. Up to 80% of the known dockerin containing subunits were identified in this study. Quantitative results show a clear pattern in regulation of cellulosomal components and their individual levels to better suit the organism's needs for growth under different conditions. While the results highlight the importance of Glycoside Hydrolase 9 family of exoglucanases and endoglucanases in degradation of plant biomass, they also point to potential bottlenecks, such as downregulation of xylanases and pectinases that may compromise the cells' ability to unwrap the intertwined polymeric compounds in plant cell walls. Such studies are vital to engineering a strain that is best suited to grow on specific substrates of interest and provide the building blocks for constructing designer cellulosomes with tailored enzyme composition for industrial ethanol production.

## Supporting Information

Table S1 (0.25 MB XLS)
